# Precision Medicine in Oncology: Imatinib Dosing in the Obese Cancer Population Using Virtual Clinical Trials

**DOI:** 10.1002/psp4.70018

**Published:** 2025-03-27

**Authors:** Khairulanwar Burhanuddin, Afzal Mohammed, Nurul Afiqah Burhanuddin, Raj K. S. Badhan

**Affiliations:** ^1^ National Pharmaceutical Regulatory Agency, Ministry of Health Malaysia Petaling Jaya Malaysia; ^2^ School of Pharmacy, College of Health and Life Science Aston University Birmingham UK; ^3^ Department of Mathematical Science, Science and Technology Universiti Kebangsaan Malaysia Bangi Selangor Malaysia

**Keywords:** cancer obesity, imatinib, PBPK, pharmacokinetics, TDM

## Abstract

This study investigates the impact of obesity on imatinib pharmacokinetics in cancer patients by utilizing physiologically based pharmacokinetic modeling (PBPK) and virtual clinical trial approaches and evaluates the effectiveness of therapeutic drug monitoring (TDM)–guided dose adjustment to recover the imatinib trough concentration (C_min_) into the target concentration. PBPK models were validated against clinical data from lean, overweight, and obese cancer populations. Simulations revealed significant physiological differences across body‐mass‐index categories, including higher body surface area, liver weight, and cardiac output in obese individuals, coupled with lower CYP3A4 enzyme activity and hematocrit levels, which translated into pharmacokinetic differences. Obese patients exhibited significantly lower imatinib maximum concentration and area‐under‐the‐curve values. C_min_ levels, a key determinant of therapeutic response, were consistently lower in the obese cohort, with a greater proportion of individuals falling below the subtherapeutic threshold (< 750 ng/mL); nevertheless, the differences are not statistically significant. TDM‐guided dose adjustments improved C_min_ levels across BMI groups. For patients with C_min_ between 450 and 750 ng/mL, dose increases of 1.5–2.0 times effectively restored levels to the target range (750–1500 ng/mL). However, individuals with C_min_ < 450 ng/mL often failed to achieve therapeutic levels, suggesting limited benefit from further dose escalation and a need for alternative therapies. This study underscores the importance of PBPK modeling and TDM in tailoring imatinib therapy for obese cancer patients by addressing physiological differences and optimizing dosing strategies for better outcomes.

**Summary**

- What is the current knowledge on the topic?
  ○ Current research shows that overweight and obese chronic myeloid leukemia (CML) patients tend to have a slower response to imatinib treatment. Higher doses of imatinib may be needed for morbidly obese patients, and studies have found lower imatinib plasma levels in heavier patients. In addition, several studies have affirmed the benefits of therapeutic drug monitoring in guiding the dose adjustment for imatinib.
- What question did this study address?
  ○ To what extent do physiological differences between lean, overweight, and obese cancer subjects influence imatinib pharmacokinetics, and can the TDM‐guided dosing titrations successfully restore imatinib trough concentrations to therapeutic levels across these populations?
- What does this study add to our knowledge?
  ○ The application of the PBPK concept in this study addresses the physiological parameter distinctness between lean, overweight, and obese cancer populations and highlights the significant variations in imatinib pharmacokinetics between the populations. Moreover, the TDM‐guided dosing strategy for imatinib effectively attained the target trough concentrations in all three population groups, particularly for subjects with trough concentrations above 450 ng/mL.
- How might this change clinical pharmacology or translational science?
  ○ The outcomes highlight the capabilities of PBPK modeling to elucidate the pharmacokinetic differences and refine the TDM dosing strategy in lean, overweight, and obese cancer populations. Continuous improvement of the virtual overweight and obese cancer populations with the latest information is imperative, with a specific focus on the physiological data that significantly influence the imatinib absorption, distribution, metabolism, and excretion.

## 1 Introduction

Obesity is recognized as a global health epidemic, with a prevalence of over 2.6 billion in 2020 and estimated to increase to over 4 billion by 2035 [1]. Furthermore, obesity is linked with an increased risk of cancer and is associated with higher cancer‐related mortality [2]. Thus, the intertwining of obesity and cancer introduces a complex mesh of physiological changes, such as metabolism enzyme activities, altered tissue composition, and others that can significantly influence drug absorption, distribution, metabolism, and excretion [3, 4, 5, 6].

Navigating the dosing approach in obese cancer patients is a conundrum. It necessitates careful consideration of how various physiological alterations in obese cancer patients can influence the pharmacokinetic parameters, such as an increase in adipose tissue composition affecting a drug with high lipophilicity, thus increasing the volume of distribution and reducing plasma concentrations [7], and the decrease in CYP3A4 metabolism enzyme abundances impacts a majority of drugs cleared through the liver [4, 8].

The advent of tyrosine kinase inhibitors (TKIs), notably imatinib, marked a breakthrough in targeted therapy for patients with chronic myeloid leukemia (CML) and gastrointestinal stromal tumor (GIST). Using a standard oral fixed‐dose regimen also poses a challenge in optimizing the dose for the obese cancer population [9, 10], suggesting a higher dose might be needed in morbidly obese cancer patients [10, 11, 12].

Data from multiple studies indicate that imatinib trough concentration (C_min_) is an excellent surrogate marker in predicting the clinical response of CML and GIST patients [13, 14]. The robust correlation between the imatinib plasma concentrations and the pharmacological effect, combined with significant inter‐patient (47%–75%) and intra‐patient variabilities (19%–30%), suits the criteria as a candidate for a therapeutic drug monitoring (TDM) approach [15, 16]. Moreover, several hospitals in Europe have implemented the TDM approach for imatinib in their clinical practice, and a consensus TDM guideline for imatinib therapy has been established by the International Association of Therapeutic Drug Monitoring and Clinical Toxicology (IATDMCT) [15, 16].

Physiologically based pharmacokinetic (PBPK) modeling capabilities to account for physiological changes and predict plasma concentrations, even with limited data, serve as a valuable tool for evaluating imatinib pharmacokinetics in special populations, such as obese cancer patients. While the modeling concept has been utilized for various drugs in obese and cancer populations individually, its application for the obese cancer population remains unexplored [4, 6]. Furthermore, the concept has also been implemented to assess the impact of TDM‐guided dose adjustment for imatinib in a Chinese cancer population [17].

Given the paucity of imatinib pharmacokinetics data in obese cancer populations, for the first time, this study has applied the PBPK concept in assessing the physiological and imatinib pharmacokinetic differences between the lean, overweight, and obese cancer populations. Additionally, the effectiveness of the TDM‐guided imatinib dose titration approach adapted from Gotta et al. (2014) [18] was evaluated in the obese cancer population.

The objectives of this study are to utilize the principle of PBPK modeling and virtual clinical trials to (1) delineate the differences in physiological parameters between lean, overweight, and obese cancer populations, (2) evaluate the influence of obesity on imatinib pharmacokinetics in adult cancer populations, and (3) assess the ability for TDM‐guided dose adjustment for imatinib to regain the sub‐ and supra‐therapeutic C_min_ into the target level.

## 2 Methods

The simulations of imatinib plasma profiles on adult cancer populations were performed using the PBPK modeling platform Simcyp Version 21 (Simcyp Ltd., a Certara company, Sheffield, UK). This study applied the four‐step workflow for validation and virtual TDM of imatinib (Figure S1, Supporting Information, Section 1).

### 2.1 Step 1: Verification of the Imatinib Model in Healthy, Cancer, and Caucasian Populations

A previously developed and validated imatinib model was used without adaptation (Table S1, Supporting Information, Section 2) [17, 19, 20]. Reverification of the imatinib model focused on reproducing pharmacokinetic parameters and plasma concentration profiles, as the model has been validated in multiple studies across diverse ethnic groups and age ranges, particularly in predicting C_min_ with significant inter‐subject variability [17, 19, 20]. It was performed with published data in three different population groups from 10 studies across six different doses (Supporting Information, Section 2).

The healthy population in Simcyp was used for simulation to verify the observed data in Group 1; the cancer population in Simcyp was utilized for verification of published data from Group 2, while the Simcyp North European Caucasian (NEurCaucasian) population was used to verify clinical data in Group 3 (Supporting Information, Section 2). The population models selected for validation align with the published data and the population model utilized by the imatinib model developer [20]. Furthermore, simulations were made with 10 trials × 10 patients designed with an age range and male percentages matching the published studies.

Three populations used to validate the imatinib model are default populations available in the Simcyp in‐house population library. The difference between the healthy adult and NEurCaucasian populations is that the NEurCaucasian population is considered a general population established from a sizable European health survey, including a population with medical conditions [20]. As for the cancer population, the main difference that influences imatinib pharmacokinetics compared to the other two populations is the higher plasma α1‐acid glycoprotein (AGP) level [6, 20].

### 2.2 Step 2: Validation of Lean, Overweight, and Obese Cancer Population With Imatinib Model

Before assessing the differences in imatinib pharmacokinetics between lean, overweight, and obese oncologic populations, we further verified the imatinib model with the Simcyp cancer population categorized according to the body mass index (BMI), lean for < 25 kg (kg)/m (m)^2^, overweight for 25 to < 30 kg/m^2^ and obese for > 30 kg/m^2^ [21].

The cancer population in Simcyp was developed based on data from patients with advanced solid tumors and generated virtual subjects with BMI from 16 to 46 kg/m^2^ [6, 8]. Within the Simcyp population library, the obese and morbidly obese populations were also available, but both were not derived based on data from cancer patients [4]. The fundamental physiological differences between cancer and obese populations in Simcyp are the plasma AGP level, CYP3A4 abundances, human serum albumin, serum creatinine, and hematocrit [4, 6].

For verification, a virtual clinical trial simulating 1000 cancer patients dosed with 400 mg daily for 28 days matches the demographic population described by Lin et al. (2023) [22]. The virtual subjects were then stratified according to their BMI categories and verified with the imatinib steady‐state C_min_ of lean, overweight, and obese cancer populations reported by Lin et al. (2023) [22] from 201 subjects (87 lean, 83 overweight, and 31 obese) aged between 24 and 88 years old.

### 2.3 Step 3: Comparison of Physiological Parameters and Imatinib Pharmacokinetics Parameters Between Lean, Overweight, and Obese Cancer Populations

For the physiological parameters comparison between lean, overweight, and obese cancer populations, 10,000 virtual cancer subjects were simulated with a 1:1 male‐to‐female ratio and aged 20–88 years. The virtual subjects were not explicitly stratified according to age, as the PBPK model accounts for physiological variations associated with aging, such as changes in renal function, enzyme activity, and body composition [6]. In addition, studies have shown no clinically significant impact of age on imatinib pharmacokinetics [13, 23].

Subsequently, we stratified the virtual subjects according to their BMI for the physiological parameters comparison, which includes height, weight, body surface area (BSA), liver weight, cardiac output, human serum albumin (HSA), hematocrit, AGP, serum creatinine, glomerular filtration rate (GFR), CYP3A4 liver enzyme abundance, CYP2C8 liver enzyme abundance, ABCB1 (P‐gp/MDR1) transporter activity, and ABCG2 (BCRP) transporter activity.

In order to identify the differences in imatinib pharmacokinetic parameters between the BMI categories, we simulated four virtual clinical trials with 1000 virtual cancer populations with an age range of 20–60 years and a 0.5 male‐to‐female ratio. For each trial, the virtual subjects with similar demographic characteristics were dosed with imatinib either at 200, 400, 600, or 800 mg daily for 28 days.

The pharmacokinetic parameters that were compared include area‐under‐the‐curve (AUC), maximum concentration (C_max_), and C_min_, all at steady state. Additionally, the percentages of C_min_ that fell outside the target range, specifically below 1100 and 750 ng/mL, as well as above 1500 ng/mL, were reported for each BMI classification. The primary target range of 750–1500 ng/mL aligns with Gotta et al. (2014) [18], while the subcriterion of 1100 ng/mL suggested by Bouchet et al. (2016) [24], was included to highlight specific therapeutic levels adopted by Lin et al. (2023) [22] whose data validated the imatinib model.

### 2.4 Step 4: Imatinib TDM in Lean, Overweight, and Obese Cancer Populations

Imatinib dose adjustment guided by TDM has been reported to improve its efficacy and has been implemented in clinical settings in numerous hospitals [23, 25]. Additionally, the concept of PBPK has been utilized as virtual TDM to assess the imatinib pharmacokinetic variabilities in the Chinese cancer population [17]. Hence, we adapted the TDM‐guided imatinib dose adjustment executed by Gotta et al. (2014) [18] (Figure S2, Supporting Information, Section 3) to evaluate the difference in imatinib pharmacokinetics between lean, overweight, and obese cancer populations using the PBPK framework.

Virtual clinical trials of 1000 adult cancer subjects (20–60 years old, 1:1 male‐to‐female ratio) dosed with imatinib 400 mg daily for 26 days were simulated, followed by dose adjustment for an additional 30 days. The C_min_ threshold window of 750–1500 ng/mL recommended by Gotta et al. (2014) [18] was fixed in this study. For each virtual trial, subjects were categorized according to their BMI: lean, overweight, and obese. This was followed by stratification according to the C_min_ after being dosed on Day‐26 (before dose adjustment). Subsequently, the ability of dose adjustment to recapitulate the subjects whose C_min_ were outside the threshold back into the target window was quantified.

### 2.5 Prediction Performance

Verification of the imatinib model with observed data in step 1 was determined by visual predictive check (VPC) and predicted/observed ratio within a two‐fold (0.5‐2‐fold) range unless otherwise explained [26, 27, 28]. The two‐fold range acceptance criteria in step 1 align with the model developer's validation criteria, though broader criteria of 2.5‐fold may be applicable for imatinib due to its high variability [29].

As for step 2, we used the VPC method alone since we only utilized the observed data reported by Lin et al. (2023) [22] to validate the imatinib model with lean, overweight, and obese cancer patients. The acceptance range for VPC is when the observed plasma concentration distribution matches the 5th and 95th percentiles of the mean simulated plasma concentration profiles [17, 19]. The 5th and 95th percentiles represent the range of uncertainty in the model predictions, providing a basis for evaluating the agreement with observed data.

All the observed data used for validation, particularly for VPC, were extracted from published studies using WebPlotDigitizer version 4.5 (https://apps.automeris.io/wpd/). Spearman's correlation test was implemented to test the correlation between C_min_ versus body weight in step 2. In addition, one‐way ANOVA with Tukey's multiple comparison tests was performed to compare the physiological and pharmacokinetic parameters between lean, overweight, and obese cancer populations in step 3. Statistical significance was set at *p* < 0.05. All the statistical analyses were run with GraphPad Prism Version 8 for Windows (GraphPad Software, La Jolla, CA, USA).

## 3 Result

### 3.1 Step 1: Imatinib Model Validation in Healthy, Cancer, and Caucasian Populations

The imatinib model was validated with three studies in healthy adults and seven studies in the adult cancer population. The pharmacokinetic parameters’ prediction‐to‐observed ratio, including C_max_, AUC_0‐inf_, AUC_0‐t_, and time‐to‐maximum concentration (T_max_), when compared with observed data from healthy adults, was between 0.72 and 1.04, within the acceptance criteria (Table 1). Furthermore, the 90% prediction intervals (5th–95th percentiles) of the simulated profiles included most of the imatinib observed concentrations, supporting the model's ability to predict imatinib plasma concentrations in healthy adults (Figure S3, Supporting Information, Section 4).

**TABLE 1:** Observed and predicted pharmacokinetic parameters of imatinib for healthy and cancer subjects.

Reference	Age, no. of subject, population	Dosing regimen	PK parameter	Observed	Predicted	Predicted/Observed
Peng et al. (2004) [48]	40–58, *n*: 12, healthy adults^a^	400 mg single‐dose	C_max_ (ng/mL)	1822 ± 1193	1669 ± 465	0.92
			AUC_0‐inf_ (ng.h/mL)	32,640 ± 16,501	23,645 ± 10,099	0.72
			T_max_ (h)	2.5 (1.0–6.0)	2.3 (1.1–3.7)	0.92
Nikolova et al. (2004) [49]	19–60, *n*: 33, healthy adults^b^	400 mg single‐dose	C_max_ (ng/mL)	1606 ± 647	1669 ± 465	1.04
			AUC_0‐24_ (ng.h/mL)	18,658 ± 8016	19,547 ± 6994	1.04
			AUC_0‐96_ (ng.h/mL)	25,150 ± 11,611	23,578 ± 10,002	0.94
			AUC_0‐inf_ (ng.h/mL)	25,464 ± 11,846	23,645 ± 10,099	0.93
			T_max_ (h)	2.5 (1.5–6.0)	2.3 (1.1–3.7)	0.92
^c^Pena et al. (2020) [50]	21–27, *n*: 26, healthy adults^c^	400 mg single‐dose	C_max_ (ng/mL)	2029 ± 551	1669 ± 465	0.82
			AUC_0‐72_ (ng.h/mL)	32,378 ± 9152	23,406 ± 9805	0.72
^d^Peng et al. (2004) [30]	53.8 ± 12.7, *n*: 3, CML patients^d^	25 mg at steady‐state	C_max_ (ng/mL)	179.9 ± 89.2	237.9 ± 84.6	1.32
			AUC_0‐24ss_ (μg.h/mL)	1.9 ± 0.9	4.0 ± 1.8	2.10
			T_max_ (h)	1.0 ± 0.5	2.4 ± 0.4	2.40
	*n*: 3, CML patients^d^	50 mg at steady‐state	C_max_ (ng/mL)	365.7 ± 75.6	475.8 ± 169.2	1.30
			AUC_0‐24ss_ (μg.h/mL)	4.6 ± 0.4	8.0 ± 3.7	1.74
			T_max_ (h)	3.8 ± 3.6	2.4 ± 0.4	0.63
	*n*: 3, CML patients^d^	85 mg at steady‐state	C_max_ (ng/mL)	799.6 ± 463.1	809.0 ± 287.8	1.01
			AUC_0‐24ss_ (μg.h/mL)	9.8 ± 3.3	13.7 ± 6.3	1.40
			T_max_ (h)	2.2 ± 1.4	2.4 ± 0.4	1.09
	*n*: 5, CML patients^d^	350 mg at steady‐state	C_max_ (ng/mL)	1407.0 ± 710.7	3335.1 ± 1186.5	2.37
			AUC_0‐24ss_ (μg.h/mL)	20.0 ± 10.6	56.2 ± 25.8	2.81
			T_max_ (h)	3.1 ± 1.0	2.4 ± 0.4	0.77
	*n*: 5, CML patients^d^	400 mg at steady‐state	C_max_ (ng/mL)	2596.0 ± 786.7	3812.2 ± 1356.4	1.47
			AUC_0‐24ss_ (μg.h/mL)	40.1 ± 15.7	64.2 ± 29.6	1.60
			T_max_ (h)	3.3 ± 1.1	2.4 ± 0.4	0.73
	*n*: 9, CML patients^d^	600 mg at steady‐state	C_max_ (ng/mL)	3508.9 ± 1649.3	5722.6 ± 2036.5	1.63
			AUC_0‐24ss_ (μg.h/mL)	51.7 ± 26.7	96.5 ± 44.4	1.87
			T_max_ (h)	3.1 ± 1.1	2.4 ± 0.4	0.77

*Note: n*, number of subjects; C_max_, maximum concentration; AUC_0‐t_, area‐under‐the‐curve to the last time point; AUC_0‐inf_, area‐under‐the‐curve extrapolated to infinity; AUC_0‐24ss_, area‐under‐the‐curve for 24 h at steady‐state; T_max_, time to maximum concentration; ^a^Compared with simulated healthy adults; ^b^Compared with simulated healthy adults; ^c^Compared with simulated healthy adults; ^d^Compared with simulated cancer subjects.

As for the adult cancer population, verification was conducted with seven studies. Only one reported [30] the pharmacokinetic parameters for comparison in addition to plasma concentration profiles (Table 1 and Figure S4, Supporting Information, Section 4), whereas the remaining reported plasma concentration profiles (Figure S3, Supporting Information, Section 4). Pharmacokinetic parameters comparison for the six doses generally adhered to the two‐fold rule except for AUC_0‐24ss_ and T_max_ at the 25 mg daily dose as well as C_max_ and AUC_0‐24ss_ at the 350 mg daily dose, where the differences were more than two‐fold (Table 1).

The predicted profiles in adult cancer populations (Figure S3, Supporting Information, Section 4) were consistent with the sparse imatinib plasma concentration data published by Petain et al. (2008) [31] (Figure S3B,C), Eechoute et al. (2012) [32] (Figure S3D,E), Renard et al. (2015) [33] (Figure S3F), and Gotta et al. (2014) [23] (Figure S3G). In general, a wider distribution was noticed in observed data compared to the 5th and 95 percentile simulated profiles for all four publications.

Overprediction was seen when a comparison was made with the steady‐state observed data from Petain et al. (2008) [31] (Figure S3C) and Peng et al. (2004) [30] (Figure S4). Nevertheless, the predictions aligned for other doses from the same study, and sparse samples from another four studies boosted the confidence of the imatinib model to predict plasma concentrations in the adult cancer population.

As for the observed imatinib concentrations reported by Widmer et al. (2006) [34] (Figure S3H) and Haouala et al. (2013) [35] (Figure S3I), the comparison was made with the simulated profiles from the NEurCaucasian population in Simcyp to match the validation method implemented by the model developer [19, 20]. The predicted profiles, including the 5th and 95th percentiles, adequately encompass the broad range of observed data.

Generally, the inter‐individual variability in observed concentrations was broader than predicted, particularly in Figure S3B,D,E (Supporting Information), reflecting a potential limitation in the model's ability to capture variability fully.

### 3.2 Step 2: Validation of Lean, Overweight, and Obese Cancer Population With Imatinib Model

**FIGURE 1:**
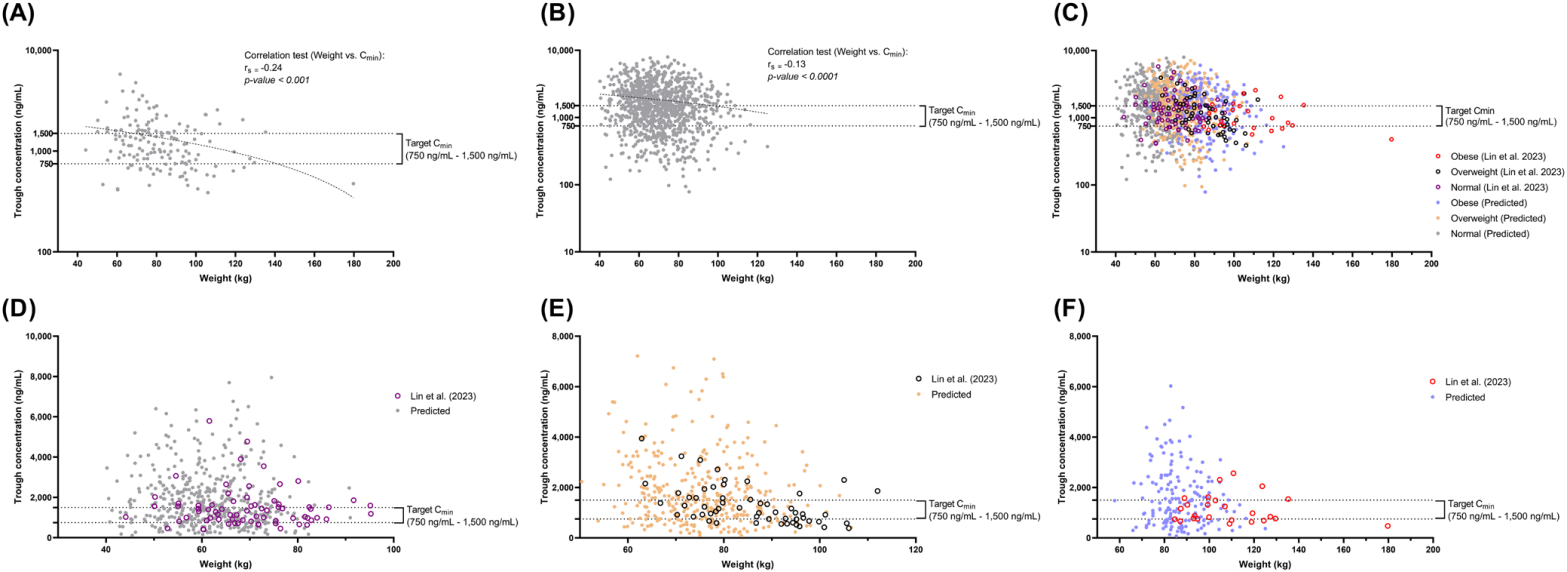
Predicted and observed imatinib C_min_ in cancer populations were stratified according to the BMI classification. (A) Correlation and regression line based on the observed imatinib C_min_ by Lin et al. (2023) [22]. (B) Correlation and regression based on the simulated imatinib C_min_. (C) Predicted versus observed imatinib C_min_ stratified according to BMI classifications. (D) Lean BMI < 25 kg/m^2^. (E) Overweight BMI 25– < 30 kg/m^2^. (F) Obese BMI > 30 kg/m^2^. Closed‐colored circles, predicted C_min_; open‐colored circles, observed C_min_.

The applicability of the imatinib model in predicting pharmacokinetic parameters of overweight and obese adult cancer populations was validated with observed C_min_ at steady‐state when cancer patients were dosed with imatinib 400 mg per day, as Lin et al. (2023) [22] reported. The reported C_min_ superimposed within the distribution of simulated C_min_ for all three populations (Figure 1C): lean (Figure 1D), overweight (Figure 1E) and obese (Figure 1F) adults.

Furthermore, the correlation test results for C_min_ versus body weight were comparable (r_s_: −0.24, *p* < 0.001 vs. r_s_:−0.13, *p* < 0.0001) between observed (Figure 1A) and simulated data (Figure 1B). Besides, the results showed that correlations between body weight and trough levels were significant in both simulated and observed imatinib concentrations. The primary difference between observed and simulated populations was the maximum body weight, with 125 kg for the virtual population and 180 kg for the actual patient.

### 3.3 Step 3: Comparison of Physiological Parameters and Imatinib Pharmacokinetics Parameters Between Lean, Overweight, and Obese Cancer Populations

The comparison of physiological attributes focused on 14 key parameters. A radar chart (Figure S5, Supporting Information, Section 5) illustrates the approximate differences between lean, overweight, and obese adult cancer populations. A substantial difference was seen in weight, BSA, liver weight, cardiac output, serum creatinine, GFR, and CYP3A4 abundance. Excluding height, hematocrit, AGP, serum albumin, and serum creatinine, the physiological parameters were higher in the obese adult cancer population as opposed to the lean adult cancer population Figure S5.

**FIGURE 2:**
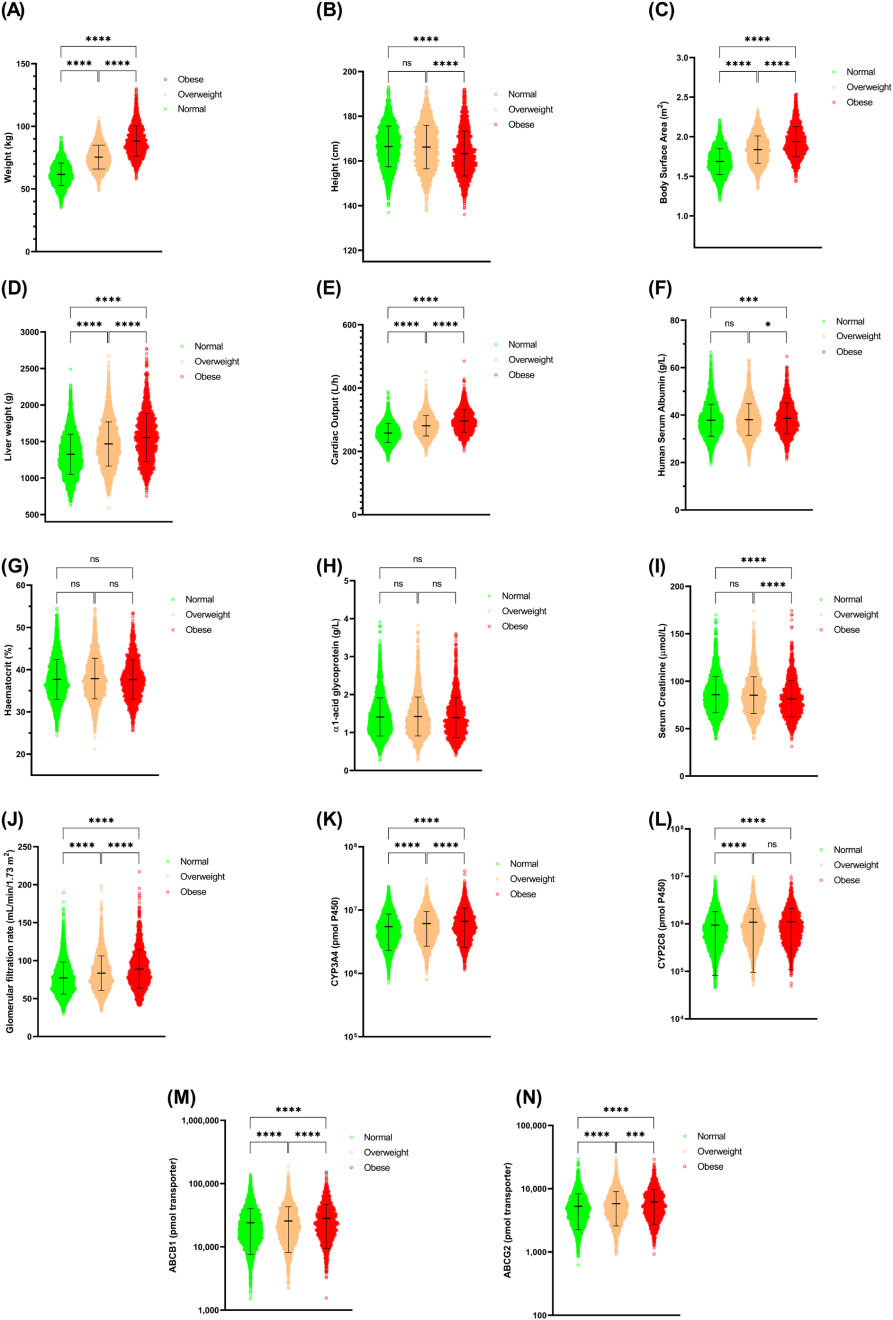
Comparison between normal, overweight, and obese adult cancer physiological parameters. Middle horizontal lines represent the mean, with upper and lower horizontal lines representing the standard deviations; *p < 0.05; ***p < 0.001; ****p < 0.0001; ns, not significant.

Furthermore, a statistical comparison test between lean, overweight, and obese adult cancer populations was performed for each physiological parameter (Figure 2). Significant differences (*p* < 0.001 for HSA and *p* < 0.0001 for other parameters) were noticed between lean and obese populations in all physiological parameters except for hematocrit and AGP (Figure 2). The differences between lean and overweight were insignificant in five out of 14 parameters, namely, height, HSA, hematocrit, AGP, and serum creatinine. In the comparison between overweight and obese populations, only hematocrit, AGP, and CYP2C8 abundance showed no significant difference, whereas the remaining parameters displayed substantial differences.

**FIGURE 3:**
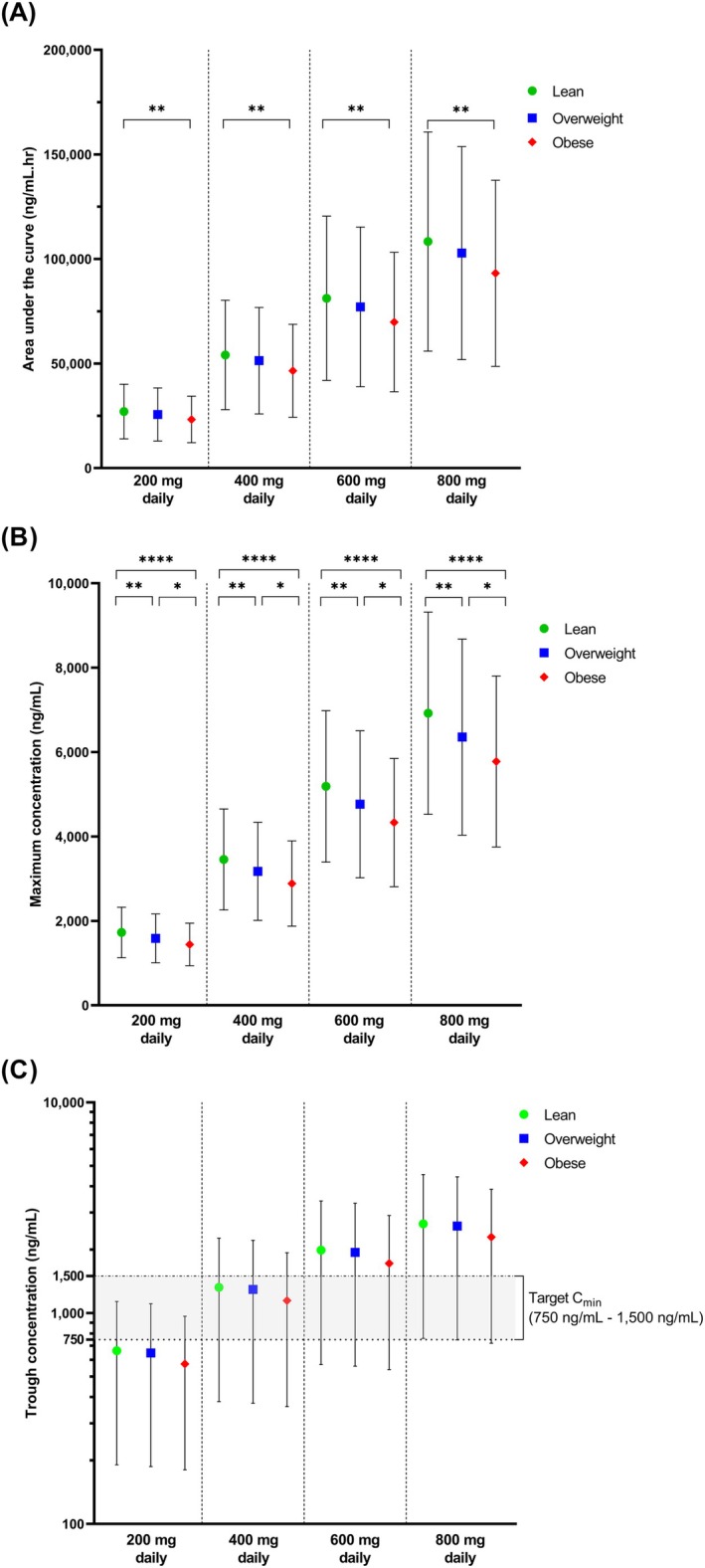
Comparison of predicted imatinib pharmacokinetic parameters at steady state between lean, overweight, and obese cancer populations. (A) Area under the curve (AUC), (B) Maximum concentrations (C_max_), (C) Trough concentrations (C_min_). *p < 0.05; **p < 0.005; ****p < 0.0001; The colored symbol represents the mean, with upper and lower horizontal lines representing the SD. The absence of a comparison bracket (horizontal bracket) on top of each mean and SD graph indicates the differences are not statistically significant.

The comparison of imatinib pharmacokinetics revealed lower AUC, C_max_, and C_min_ in obese adult cancer populations compared to lean adults, with statistically significant differences observed for AUC and C_max_ but not for the C_min_ (Table 2). The same trend was seen across all four doses (200–800 mg daily). Multiple comparisons between lean, overweight, and obese adult cancer populations revealed that the differences were significant between each population for the C_max_ parameter, while for AUC, a notable difference was displayed only between lean and obese populations (Figure 3). With respect to the C_min_, the difference between the populations was not statistically significant (Figure 3).

**TABLE 2:** Comparison of simulated imatinib pharmacokinetic parameters at steady state between lean, overweight, and obese cancer populations.

Dose	Body mass index (BMI) status	PK parameters			Percentage of subjects with C_min_		
		AUC_0–24_ (ng/mL.h)	C_max_ (ng/mL)	C_min_ (ng/mL)	< 1100 ng/mL	< 750 ng/mL	> 1500 ng/mL
200 mg daily	Normal (*n* = 474)	27,050.45 ± 13,070.92	1726.66 ± 597.63	663.17 ± 472.46	84.81	66.46	6.12
	Overweight (*n* = 359)	25,677.83 ± 12,706.12	1585.76 ± 579.84	647.58 ± 460.43	85.24	69.36	6.13
	Obese (*n* = 167)	23,266.19 ± 11,103.62	1441.88 ± 505.74	573.96 ± 393.50	91.02	6.13	3.59
	*p*	0.004	< 0.0001	0.09			
400 mg daily	Normal (*n* = 474)	54,112.87 ± 26,166.38	3455.19 ± 1195.81	1326.01 ± 945.59	51.69	31.65	33.54
	Overweight (*n* = 359)	51,378.04 ± 25,429.22	3173.40 ± 1160.88	1295.36 ± 921.61	52.09	32.31	30.92
	Obese (*n* = 167)	46,537.47 ± 22,225.62	2885.32 ± 1011.40	1147.28 ± 787.42	55.09	38.32	25.75
	*p*	0.004	< 0.0001	0.09			
600 mg daily	Normal (*n* = 474)	81,217.85 ± 39,271.51	5187.34 ± 1795.56	1989.59 ± 1419.27	31.22	15.61	53.16
	Overweight (*n* = 359)	77,100.91 ± 38,162.84	4763.74 ± 1741.37	1943.34 ± 1382.84	31.75	17.55	53.48
	Obese (*n* = 167)	69,842.12 ± 33,352.38	4330.59 ± 1518.87	1721.86 ± 1182.03	35.93	21.56	50.30
	*p*	0.004	< 0.0001	0.09			
800 mg daily	Normal (*n* = 474)	108,336.71 ± 52,396.87	6921.25 ± 2395.50	2653.12 ± 1893.45	17.72	10.13	68.35
	Overweight (*n* = 359)	102,844.67 ± 50,911.82	6355.48 ± 2322.86	2591.45 ± 1844.72	21.45	11.14	67.69
	Obese (*n* = 167)	93,158.16 ± 44,494.66	5777.57 ± 2026.19	2295.98 ± 1576.86	26.35	14.37	61.68
	*p*	0.004	< 0.0001	0.09			

*Note:* Mean ± SD; PK, pharmacokinetic; AUC_0‐24_, area‐under‐the‐curve from 0 to 24 h; C_max_, maximum concentration; C_min_, trough concentration; *p < 0.05*, statistically significant difference; C_min_ < 1100 ng/mL is the C_min_ threshold proposed by Bouchet et al. (2016) [24]; C_min_ < 750 ng/mL and > 1500 ng/mL are the lower and upper threshold for C_min_ suggested by Buclin et al. (2020) [16].

In relation to the lower value of C_min_ in the obese population, the percentage of subjects with C_min_ below 1100 and 750 ng/mL was higher in the obese population compared to the overweight and lean populations (Table 2). On the other hand, the percentage was lower for the number of subjects with C_min_ above 1500 ng/mL in the obese adult cancer population (Table 2). When the virtual subjects were dosed with a standard starting dose of 400 mg daily, approximately 50% of subjects across all three BMI categories had C_min_ below 1100 ng/mL, with differences ranging from 0.4% to 3.4%. Meanwhile, the percentage of subjects with C_min_ below 750 ng/mL showed wider variation across lean, overweight, and obese populations, ranging from 0.66% to 6.67%. The results of the pharmacokinetic comparison were similar across all four doses.

### 3.4 Step 4: Virtual TDM of Imatinib in Lean, Overweight, and Obese Cancer Populations

An investigation on the effectiveness of TDM‐guided dose adjustment to recapitulate the out‐of‐range C_min_ into the target window of 750–1500 ng/mL, with virtual subjects stratified according to their C_min_ level, revealed no significant difference in the mean of C_min_ between the lean, overweight, and obese populations (*p* > 0.05) across all six C_min_ levels (Table 3), complementing the results when a comparison was made without stratification.

**TABLE 3:** Predicted imatinib trough plasma concentrations at different doses in cancer subjects following the application of TDM.

Pre‐adjustment				Post‐adjustment			
C_min_ level (ng/mL)	BMI Status	C_min_ (ng/mL)	Subjects within target C_min_ (% (proportion))	Dose adjustment	Adjusted Dose (mg)	C_min_ (ng/mL)	Subjects within target C_min_ (% (proportion))
< 450	Lean	292.79 ± 94.93	12.87 (61/474)	x 1.75	700	510.65 ± 165.03	4.91 (3/61)
				x 2	800	583.68 ± 188.84	21.31 (13/61)
	Overweight	290.63 ± 98.39	14.21 (51/359)	x 1.75	700	506.15 ± 171.81	3.92 (2/51)
				x 2	800	578.36 ± 196.32	21.57 (11/51)
	Obese	274.79 ± 108.82	18.56 (31/167)	x 1.75	700	478.63 ± 190.18	9.68 (3/31)
				x 2	800	546.85 ± 217.31	22.58 (7/31)
450–549	Lean	491.47 ± 30.15	4.85 (23/474)	x 1.5	600	736.58 ± 45.64	43.48 (10/23)
				x 1.75	700	859.05 ± 53.13	100 (23/23)
				x 2	800	981.51 ± 60.61	100 (23/23)
	Overweight	502.82 ± 19.69	6.96 (25/359)	x 1.5	600	751.61 ± 29.24	56.00 (14/25)
				x 1.75	700	876.72 ± 34.18	100 (25/25)
				x 2	800	1001.80 ± 39.13	100 (25/25)
	Obese	506.76 ± 31.42	7.19 (12/167)	x 1.5	600	756.76 ± 47.98	58.33 (7/12)
				x 1.75	700	882.83 ± 55.95	100 (12/12)
				x 2	800	1008.89 ± 63.93	100 (12/12)
550–649	Lean	592.90 ± 25.11	6.12 (29/474)	x 1.25	500	738.27 ± 30.94	34.48 (10/29)
				x 1.5	600	885.70 ± 37.14	100 (29/29)
				x 1.75	700	1033.21 ± 43.33	100 (29/29)
				x 2	800	1180.65 ± 49.58	100 (29/29)
	Overweight	602.03 ± 30.39	5.57 (20/359)	x 1.25	500	750.71 ± 37.71	55 (11/20)
				x 1.5	600	900.76 ± 45.37	100 (20/20)
				x 1.75	700	1050.79 ± 53.04	100 (20/20)
				x 2	800	1200.78 ± 60.77	100 (20/20)
	Obese	589.15 ± 30.01	5.39 (9/167)	x 1.25	500	734.25 ± 41.31	33.33 (3/9)
				x 1.5	600	881.00 ± 49.69	100 (9/9)
				x 1.75	700	1027.73 ± 58.07	100 (9/9)
				x 2	800	1174.40 ± 66.49	100 (9/9)
650–749	Lean	695.11 ± 28.87	7.81 (37/474)	x 1.25	500	867.20 ± 35.70	100 (37/37)
				x 1.5	600	1040.55 ± 42.73	100 (37/37)
				x 1.75	700	1213.90 ± 49.93	100 (37/37)
				x 2	800	1387.05 ± 56.99	100 (37/37)
	Overweight	691.57 ± 25.07	5.29 (19/359)	x 1.25	500	862.38 ± 28.92	100 (19/19)
				x 1.5	600	1034.65 ± 34.73	100 (19/19)
				x 1.75	700	1206.87 ± 40.56	100 (19/19)
				x 2	800	1379.05 ± 46.37	100 (19/19)
	Obese	699.23 ± 31.97	6.59 (11/167)	x 1.25	500	875.70 ± 40.74	100 (11/11)
				x 1.5	600	1050.03 ± 48.34	100 (11/11)
				x 1.75	700	1224.37 ± 56.03	100 (11/11)
				x 2	800	1398.72 ± 63.81	100 (11/11)
750–1500	Lean	1071.18 ± 205.68	34.39 (163/474)	None	400		
	Overweight	1094.25 ± 209.29	37.05 (133/359)				
	Obese	1127.23 ± 225.99	36.53 (61/167)				
> 1500	Lean	2382.12 ± 847.35	33.97 (161/474)	x 0.75	300	1783.48 ± 635.32	44.10 (71/161)
				x 0.5	200	1188.79 ± 423.38	80.75 (130/161)
				x 0.25	100	594.30 ± 211.61	18.01 (29/161)
	Overweight	2415.57 ± 798.15	30.92 (111/359)	x 0.75	300	1808.56 ± 597.80	37.84 (42/111)
				x 0.5	200	1205.51 ± 398.41	79.28 (88/111)
				x 0.25	100	602.65 ± 199.13	19.82 (22/111)
	Obese	2227.00 ± 616.96	25.75 (43/167)	x 0.75	300	1667.83 ± 462.73	53.49 (23/43)
				x 0.5	200	1111.72 ± 308.34	86.05 (37/43)
				x 0.25	100	555.81 ± 154.09	13.95 (6/43)

*Note:* C_min_, trough concentration; Shaded row represent the target C_min_; target C_min_, 750–1500 ng/mL.

Abbreviations: BMI, body mass index; TDM, therapeutic drug monitoring.

**FIGURE 4:**
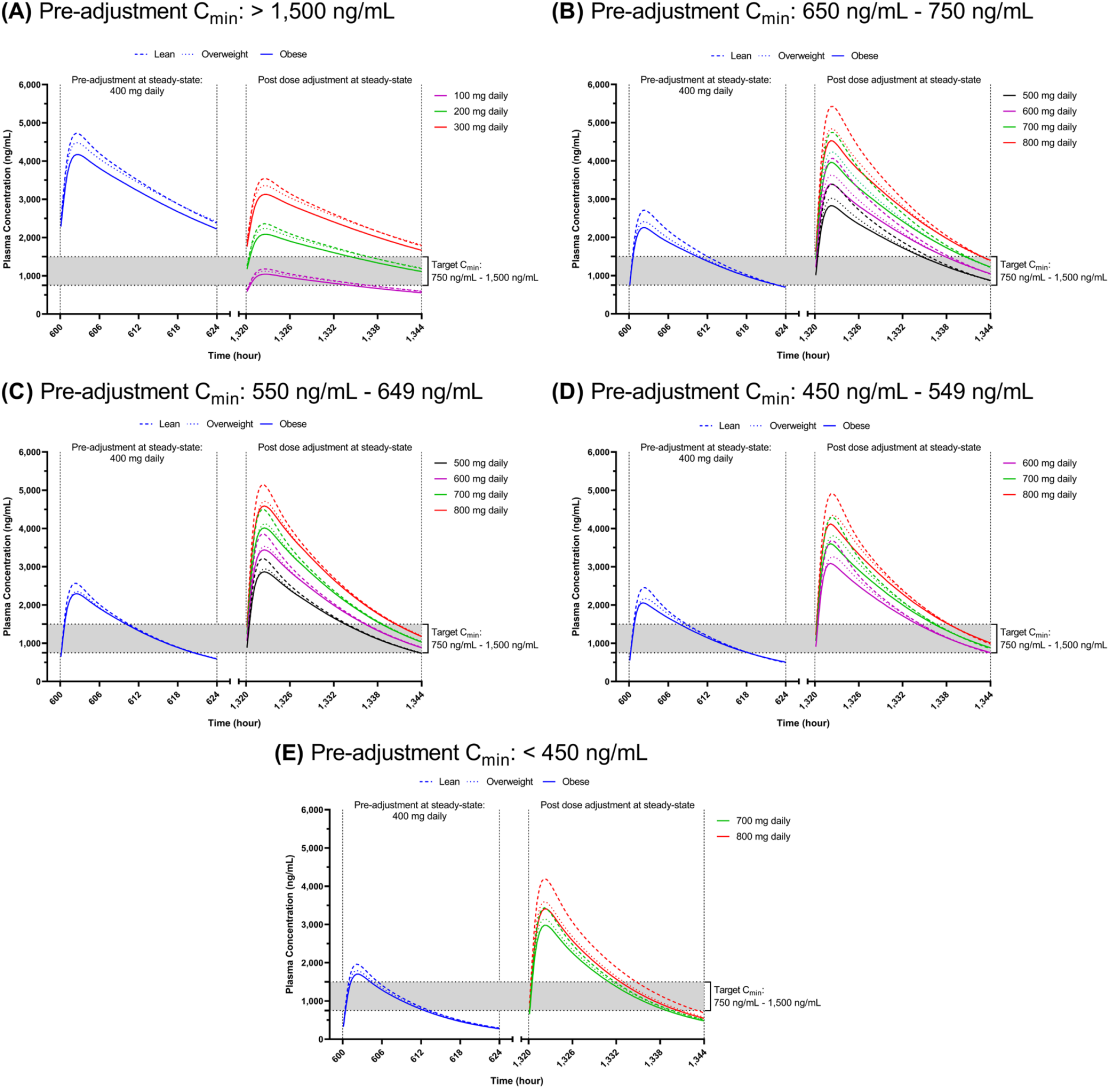
Application of therapeutic drug monitoring on the predicted imatinib plasma concentration profiles in lean, overweight, and obese cancer populations. Plasma profiles were stratified according to the trough concentration (C_min_) level: (A) > 1500 ng/mL; (B) 650–750 ng/mL; (C) 550–649 ng/mL; (D) 450–549 ng/mL; (E) < 450 ng/mL. Line patterns represent the body mass index (BMI) classification. Line colors represent the dose. The shaded area is the target C_min_ concentration.

From another perspective, the percentage of subjects with C_min_ below 550 ng/mL was highest in the obese cancer population, followed by overweight and lean (25.75%, 21.17% and 17.72%). Nevertheless, for virtual subjects with C_min_ less than 450 ng/mL, despite a two‐fold increase in dose, only approximately 20% of subjects achieved the target threshold of 750 ng/mL (Table 3), with the mean C_min_ not reaching the target window (Figure 4) for all three populations. On the other hand, for the group with C_min_ between 450 and 549 ng/mL, an increment of 1.75‐fold (700 mg daily) or two‐fold (800 mg daily) raised all the C_min_ to the target level for all three populations (Figure 4). However, with a 1.5‐fold dose increase (600 mg daily), the C_min_ of approximately 55% of overweight and obese populations ascended to the target window, but only 43.48% for the lean population.

In general, any increment between 1.25 fold (500 mg daily) and two fold (800 mg daily) raised all the subjects with a trough between 650 and 750 ng/mL up to above 750 ng/mL, while only a 1.5‐fold (600 mg daily) to two‐fold (800 mg daily) increase in dose was needed to bring up all the troughs between 550 and 649 ng/mL, beyond the 750 ng/mL target level (Table 3). For the 550–649 ng/mL stratified group, an increment of 100 mg daily only raised 33.33%–55% of the C_min_ for all three populations above the target of 750 ng/mL.

For virtual subjects with C_min_ above 1500 ng/mL, a 50% dose reduction (200 mg daily) across all three populations managed to lower the C_min_ within the aimed level of 750–1500 ng/mL (Figure 4). In terms of percentages, by reducing the standard dose by 50%, C_min_ was reduced to within 750 and 1500 ng/mL for approximately 80% of subjects with C_min_ above 1500 ng/mL, with the obese population reaching up to 86.05% (Table 3). For a dose reduction of 0.75‐fold (300 mg daily), the trough mean for all three populations was still above 1500 ng/mL. Contrarily, with a 0.25‐fold dose reduction (100 mg daily), the mean of C_min_ for all the populations falls below the 750 ng/mL threshold.

When considering higher C_min_ threshold levels, particularly > 1000 ng/mL for CML patients and > 1100 ng/mL for GIST patients, along with the simulated mean of C_min_ based on the stratified group, the imatinib dose titration guided by C_min_ remains consistent across the lean, overweight, and obese cancer populations.

## 4 Discussion

Several studies have suggested that overweight and obese patients treated with imatinib have lower responses compared with lean patients from the pharmacodynamic perspectives, such as time to achieve major molecular response (MMR), complete cytogenetic response (CCyR), overall objective benefit rate (OOBR), and time to progression (TTP) [11, 12, 14]. In this study, we take advantage of the PBPK concept to investigate the pharmacokinetic difference between lean, overweight, and obese adult cancer populations and explore the TDM approach in establishing the dose adjustment scheme for effective imatinib treatment outcomes within the pharmacokinetic aspect.

The verification of the imatinib PBPK model in healthy subjects demonstrated its ability to predict imatinib plasma profiles and pharmacokinetics parameters within acceptable margins compared to the observed data. These results are consistent with previous publications employing the same imatinib models [17, 19, 20].

Concerning the cancer patient population, verification focused on five studies involving GIST and CML patients. Imatinib exhibits high inter‐ and intra‐individual variability in pharmacokinetic parameters, which were reflected in the imatinib observed data. Generally, the model was able to capture the variability of plasma concentration data across all five studies, particularly those that reported sparse samples, further supporting the model's robustness [23, 24, 25, 30, 32, 34, 36]. Overestimation for a few simulations is possibly due to the lower number of patients in the reported data alongside the large inter‐ and intra‐individual variability of imatinib, particularly when a comparison was made with observed data from Peng et al. (2004) [25, 30].

The model also demonstrated predictive capability in patients stratified by body weight. According to existing literature, only one study [22] (to date) has reported imatinib C_min_ in lean, overweight, and obese patients with GIST (*n* = 200) and dermatofibrosarcoma (*n* = 1). The model successfully reproduced the observed pattern of body weight –C_min_ correlation, verifying its applicability across weight and BMI ranges.

A slight deviation in the regression line trends between observed and predicted C_min_ across body weight in terms of the on‐target concentrations was potentially due to the limited number of observed data points for overweight and obese patients. Moreover, the Simcyp cancer population model used for simulations was limited to a maximum weight of 125 kg, whereas the observed data included patients weighing up to 180 kg. Extending simulations to higher weights could improve predictive accuracy, particularly on the correlations, though challenges remain due to the lack of validated physiological data for morbidly obese cancer populations.

Although the imatinib model demonstrated adequate performance under the predefined validation criteria, certain limitations warrant consideration. For example, broader variability in observed data and overestimation in some outcome simulations suggest potential areas for refinement. Importantly, model parameters were not re‐estimated to maintain alignment with previous validations across diverse populations and ethnicities [17, 19, 20]. Nevertheless, further refinements may focus on incorporating additional mechanistic insights, such as hepatic enzyme activity and transporter expression, to address the variability while maintaining alignment with the existing model.

It is also worth noting that the VPC approach, though well‐established in Population Pharmacokinetics (PopPK) modeling, its application in PBPK studies remains relatively adaptable. Unlike PopPK models, which statistically estimate variability from observed data, PBPK models integrate a mechanistic understanding of physiological processes and biological variability [37]. As such, VPC in PBPK modeling assesses the alignment between observed data and predictions based on these mechanistic frameworks, which underscores the importance of careful interpretation of VPC results, particularly when applied to diverse and complex patient populations.

Differences in physiological parameters between obese and lean patients are expected due to larger body weight, higher BSA, and distinct liver size and cardiac output based on BSA [4, 38]. For a full analysis and interpretation of these outcomes, please see Supporting Information, Section 6.

In terms of imatinib pharmacokinetics differences between lean, overweight, and obese cancer populations, the differences in C_max_ resulted from variations in BSA, CYP enzymes, and transporters [12]. Imatinib's lipophilic nature increases its distribution in obese patients, lowering C_max_ and contributing to higher clearance [7]. The AUC difference is notable between lean and obese populations, while C_min_ is lower in obese groups but not statistically significant and is attributed to plasma protein binding, CYP3A4 enzymes, and transporter expression, confirmed by sensitivity analysis (Supporting Information, Section 7) [16, 18, 30].

Simulation of TDM‐guided dose adjustment strategy based on Gotta et al. (2014) [18] yielded consistent outcomes across BMI categories by successfully restoring C_min_ between 450 and 750 ng/mL to the target range of 750–1500 ng/mL. These findings align with previous reports, advocating TDM to optimize imatinib treatment outcomes, particularly in obese cancer patients [10, 22, 39]. Indeed, imatinib TDM approaches have been shown to recover and maintain more than 90% of the patient's C_min_ with a subtherapeutic range back to the target level [36].

The simulated dose reduction strategy effectively recovered most subjects with C_min_ above the upper threshold into the target range, particularly with a 25%–50% dose reduction, especially for obese cancer subjects. These results align with clinical studies indicating that dose reduction from 400 mg to 200–300 mg daily due to adverse events can still demonstrate effective outcomes while minimizing toxicity [40].

Simulations showed that only 20% of subjects with imatinib C_min_ below 450 ng/mL could reach the target level, even at 800 mg daily. Based on this data and the correlation between imatinib C_min_ with MMR and CCyR, a suboptimal response may be expected in this group of subjects due to the inability to achieve the minimum threshold of C_min_ even with the maximum dose of imatinib [41, 42]. Imatinib resistance, either due to *BCR‐ABL* dependence or independence, can be speculated to be the reason for the treatment failure [43]. Therefore, switching to an alternative therapy, such as other TKIs, at the early stage of treatment can potentially offer therapeutic advantages to the patients [42, 44, 45].

PBPK modeling offers distinct advantages for TDM in drugs with fixed‐dose regimens and limited dose adjustment flexibility, such as imatinib. It provides a simplified and practical dose adjustment framework to assist physicians in making decisions. Nevertheless, PopPK modeling remains indispensable in the TDM setting, particularly for drugs requiring individualized dosing, as it leverages measured concentration data along with covariate inputs to enable precise dose refinements.

Currently, the TDM approach for imatinib is accessible to patients in several hospitals located in European regions [16]. Nevertheless, the obstacles to widespread implementation persist, ranging from constraints associated with precise sampling time points, prescribers' hesitancy to deviate from existing dosing approaches, insufficient support for interpreting concentration into a dosing recommendation, and limitations on the bioanalytical technique [16]. Advances in bioanalytical methods such as dried blood spot analysis and accessibility of various user‐friendly software with artificial intelligence are believed to revolutionize the TDM practice and captivate prescribers' interest [46, 47].

## Author Contributions

K.B., A.M., N.A.B., and R.K.S.B. wrote the manuscript. K.B. and R.K.S.B. designed the research. K.B. and R.K.S.B. performed the research. K.B. analyzed the data.

## Consent

The institutional review board approval and informed consent are not required as no patients were recruited for this study.

## Conflicts of Interest

The authors declare no conflicts of interest.

## Supporting information


Data S1.
