# Supplementary material for: Precision Medicine in Oncology: Imatinib Dosing in the Obese Cancer Population Using Virtual Clinical Trials
Source: CPT Pharmacometrics Syst Pharmacol. 2025 Mar 27;14(6):1050–64. doi: 10.1002/psp4.70018 (PMC12167919; doi:10.1002/psp4.70018)
Supplement: Supplementary file 1 — Data S1. [file PSP4-14-1050-s001.docx]

**Precision Medicines in Oncology: Imatinib Dosing in the Obese Cancer Population using Virtual Clinical Trials**

Khairulanwar Burhanuddin^a,b^, Afzal Mohammed^b^, Nurul Afiqah Burhanuddin^c^, and Raj K. S. Badhan^b^

^a^National Pharmaceutical Regulatory Agency, Ministry of Health Malaysia, Petaling Jaya, Malaysia

^b^School of Pharmacy, College of Health and Life Science, Aston University, Birmingham, B4 7ET, United Kingdom

^c^Department of Mathematical Science, Faculty of Science and Technology, Universiti Kebangsaan Malaysia, 43600 UKM Bangi, Selangor, Malaysia

# Supplementary Materials

# Section 1: Workflow model


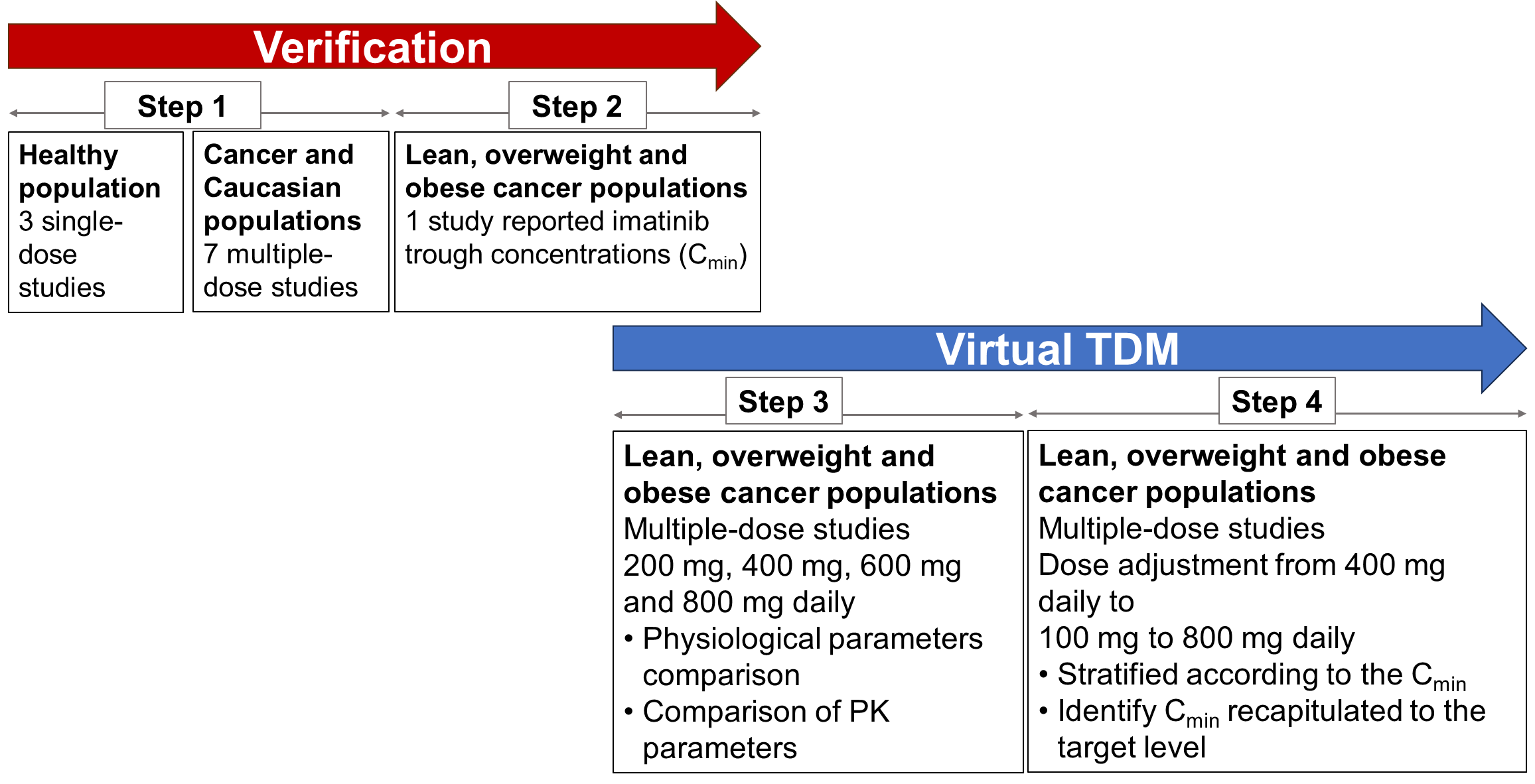


**Figure S1.** Four-step workflow model for Imatinib model verification, TDM and exploration

# Section 2: Population groups used to validate the imatinib compound model

Group 1 were healthy adults administered 400 mg as a single dose in (i) 12 adults (40 – 58 years old),^1^ (ii) 33 adults (19 – 60 years old)^3^ and (iii) 26 adults (21 – 27 years old);^4^ Group 2 were adult cancer patients administered 400 mg daily in (iv) 34 GIST patients (28 – 84 years old),^5^ (v) 50 GIST cancer subjects (39 – 82 years old),^6^ (vi) 103 pulmonary arterial hypertension (PAH) patients (18 – 77 years old),^7^ (vii) 2,478 CML patients (18.3 – 91.5 years old)^8^ and (xiii) imatinib dosed over 25 mg to 600 mg daily in 64 CML patients (53.8 ± 12.7 years old);^2^ Group 3 were Caucasian adults administered 400 mg daily in (ix) 59 CML and GIST Caucasian patients (20 – 79 years old)^9^ and (x) 49 GIST Caucasian patients (24 – 88 years old).^10^

**Table S1.** Validated imatinib compound parameter values used for simulation

| **Parameters** | **Values** | **Notes** |
| --- | --- | --- |
| **Physical chemistry and blood binding** | | |
| Compound type | Diprotic base |  |
| Molecular weight (g/mol) | 493.6 | Drug Bank^11^ |
| Log P | 1.99 | Peng et al. (2005)^12^ |
| pKa 1;2 | 8.07; 3.73 |  |
| B/P | 0.73 | Kretz et al. (2004)^13^ |
| fu | 0.05 | Smith et al. (2004)^14^ |
| Plasma binding component | α_1_-acid glycoprotein |  |
| **Absorption** | | |
| Model | ADAM |  |
| f_uGut_ | 1.00 |  |
| Q_Gut_ (L/h) | 6.04 |  |
| P_eff_ in man (10^-4^ cm/s) | 0.92 |  |
| **Distribution** | | |
| Model | Full PBPK |  |
| V_ss_ (L/kg) | 1.80 | Predicted using Method 2 by Rogers and Rowland approach^15^ |
| Kp scalar |  |  |
| **Elimination** | | |
| Model | Enzyme kinetics |  |
| Pathway 1 | CYP3A4 | N-desmethyl imatinib formation |
| V_max_ (pmol/min/pmol isoform) | 3.00 |  |
| K_m_ (µmol/L) | 10.54 |  |
| fu_mic_ | 0.96 |  |
| Pathway 2 | CYP2C8 | N-desmethyl imatinib formation |
| V_max_ (pmol/min/pmol - isoform) | 56.40 |  |
| K_m_ (µmol/L) | 7.49 |  |
| fu_mic_ | 0.97 |  |
| Pathway 3 | CYP3A4 | Other metabolites |
| CL_int_ (µl/min/mg - protein) | 33.40 |  |
| fu_mic_ | 1.00 |  |
| Pathway 4 | CYP2C8 | Other metabolites |
| CL_int_ (µl/min/mg - protein) | 24.20 |  |
| fu_mic_ | 1.00 |  |
| HLM CL_int_ (µL/min/mg - protein) | 31.00 |  |
| CL_R_ (L/h) | 0.50 | Bornhauser et al. (2005)^16^ |
| **Drug transport – hepatobiliary transporter** | | |
| Pathway 1 | ABCB1 |  |
| CL_int_,_T_ (µl/min/million - cells) | 1.50 |  |
| RAF | 1.00 |  |
| Pathway 2 | ABCG2 |  |
| J_max_ (pmol/min/million – cells) | 89.40 |  |
| K_m_ (µmol/L) | 4.37 |  |
| RAF | 0.38 |  |
| CL_PD_ (ml/min/million – hepatocytes) | 0.20 |  |
| **Drug interactions** | | |
| CYP3A4 |  |  |
| Competitive inhibition |  |  |
| K_i_ (µmol/L) | 14.30 |  |
| fu_mic_ | 0.80 |  |
| Mechanism-based inhibition |  |  |
| k_inact_ (1/h) | 4.29 |  |
| fu_mic_ | 0.80 |  |
| The compound data was adopted from Adiwidjaja et al. (2019) ^17^; Log P, partition coefficient; B/P, blood-to-plasma ratio; fu, unbound fraction; ADAM, Advance dissolution, absorption and metabolism; P_eff_, human jejunum effective permeability; PSA, polar surface area; HBD, number of hydrogen bond donors; f_uGut_, unbound fraction of drug in enterocytes; V_ss_, steady-state volume of distribution; Kp scalar, tissue partition coefficient; HLM CL_int_, human liver microsomes invitro intrinsic clearance; CL_R_, renal clearance; V_max_, maximum rate of metabolism; K_m_, Michaelis-Menten constant; fu_mic_, fraction of unbound drug in the in vitro microsomal incubation; CL_int_, in vitro intrinsic clearance; CL_PD_, passive diffusion clearance; CL_int,T_, in vitro transporter-mediated intrinsic clearance; J_max_, in vitro maximum rate of transporter-mediated efflux or uptake; RAF, relativity activity factor; K_i_, the concentration of inhibitor that supports half-maximal inhibition; k_inact_, inactivation rate of the enzyme. | | |

# Section 3: Imatinib Therapeutic Drug Monitoring (TDM) process


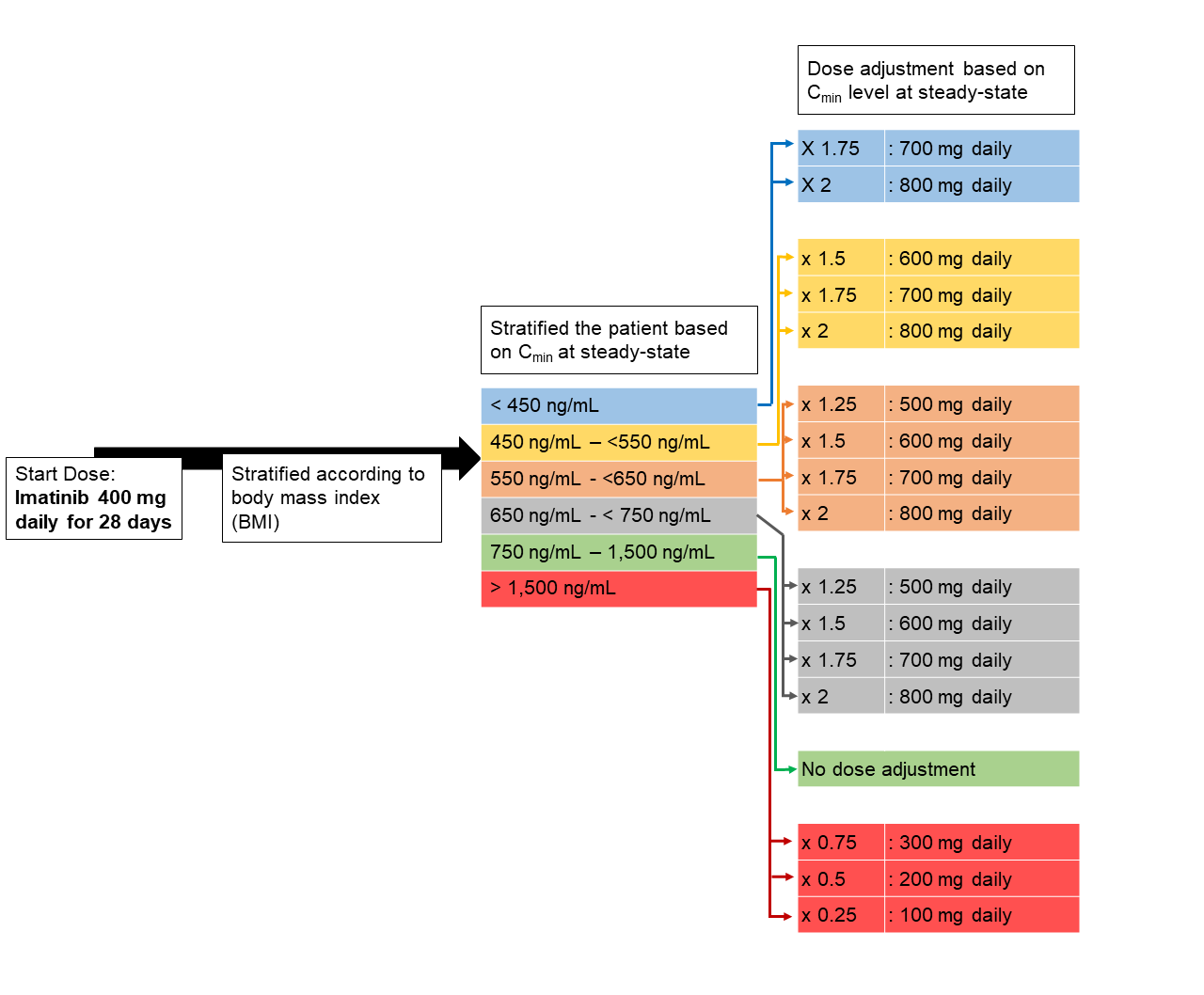


**Figure S2**. Imatinib dose adjustment guided by TDM process

# **Section 4: Results Step 1**





**Figure S3**. Comparison of model prediction and observed plasma concentration data of imatinib in healthy adults (A), adult cancer (B – G) and European adults (H-I). Solid lines represent the mean predicted concentration-time profile, with dotted lines representing the 5^th^ and 95^th^ percentile ranges. Solid circles represent observed clinical data from each study. (A) 400 mg single-dose in healthy adults;^1,3,4^ (B) 400 mg day-1 and (C) 400 mg at steady-state in cancer populations compared to Petain et al. (2008)^5^ observed data; (D) 400 mg day 1 and (E) 400 mg daily at steady-state in cancer populations compared with published data by Eechoute et al. (2012);^6^ (F) 400 mg daily at steady-state in pulmonary arterial hypertension populations compared with Renard et al. (2015)^7^ observed data; (G) 400 mg daily at steady-state in cancer populations compared with Gotta et al. (2014)^8^ observed data; 400 mg daily at steady-state in European populations compared with observed data by Widmer et al. (2006)^9^ (H) and Haouala et al. (2013)^10^ (I).


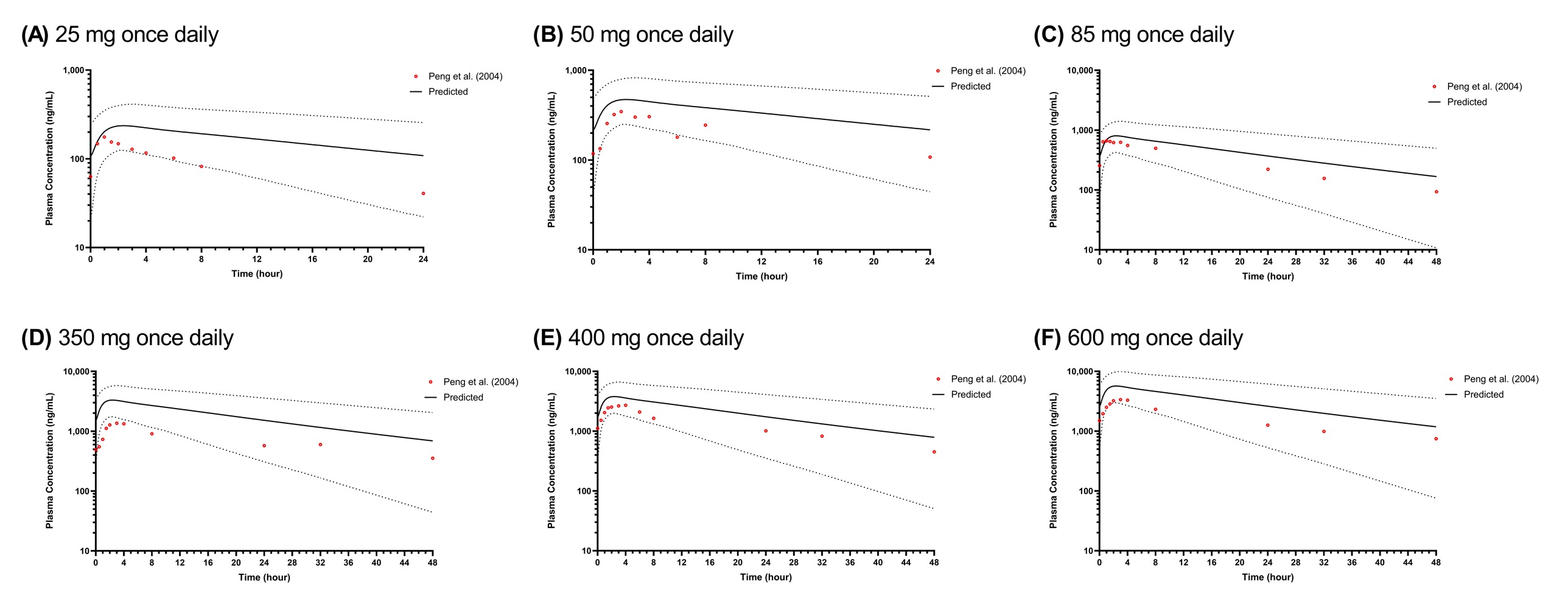


**Figure S4.** Comparison of imatinib simulated profiles with observed plasma profiles at steady-state in cancer populations. Solid lines represent the mean predicted concentration-time profile, with dotted lines representing the 5^th^ and 95^th^ percentile ranges. Solid circles represent observed clinical data from each study. Virtual cancer patients were administered with 25 mg once daily (A), 50 mg once daily (B), 85 mg once daily (C), 350 mg once daily (D), 400 mg once daily (E), and 600 mg once daily (F). Observed profiles were published by Peng et al. (2004).^2^

# Section 5: Results Step 3


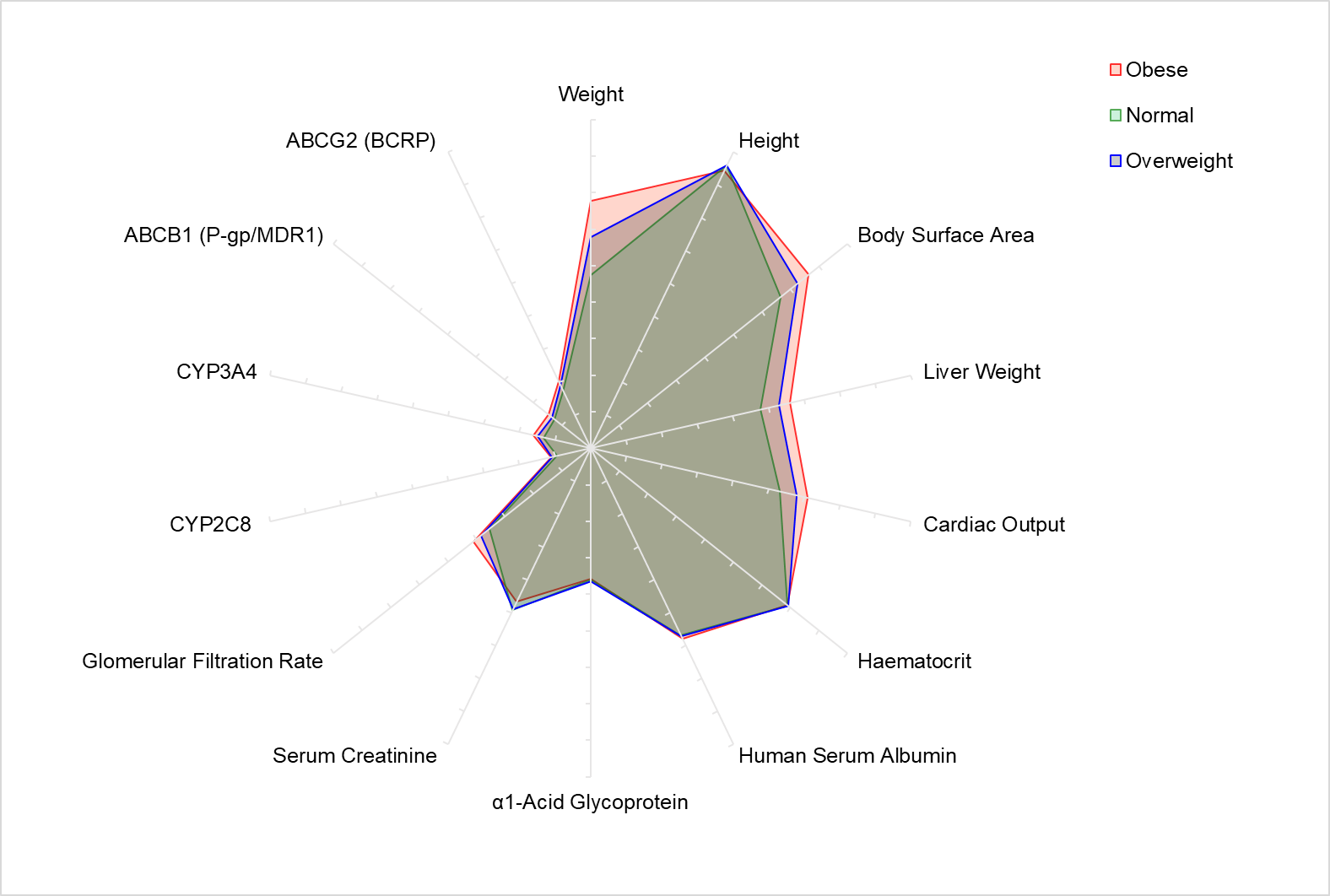


**Figure S5.** Radar graph comparing the mean of normal, overweight and obese adult cancer physiological parameters. Each colour line represents the mean for each physiological parameter, with the red line as ‘obese cancer population’, the green line as ‘lean cancer population’ and the blue line as ‘overweight cancer population’. The length of each axis represents the maximum value of the parameters, and the connected point for each line represents the mean of each population.

# Section 6: Summary analysis of physiological parameters across lean, overweight, and obese cancer populations

The differences for most parameters were also seen between lean and overweight, as well as overweight and obese cancer populations. Liver size and cardiac output were predicted based on the body surface area (BSA), hence the distinctive difference between lean, overweight and obese populations.^18^ Besides, higher cardiac output is attributed to elevated heart rate and higher stroke volume in the obese population.^19^

The prevalence of impaired renal function is higher in cancer patients, as represented by glomerular filtration rate (GFR) measurement and estimated from the serum creatinine, which has an inverse relationship between serum creatinine and GFR.^20,21^ From the body mass index (BMI) perspective, higher GFR was seen in higher BMI patients, further explaining the significant differences between all three populations.^22,23^ Since imatinib and its metabolites are mainly excreted via the faeces, with approximately 68% and 13% via the urine, the variation in renal function between the three groups may not influence the imatinib pharmacokinetics.^12^

The cytochrome P450 (CYP) enzymes and transporters that play a major contribution in imatinib metabolism and efflux include CYP3A4, CYP2C8, ABCB1 (P-gp/MDR1) and ABCG2 (BCRP).^24,25^ Due to this fact, the related enzymes and transporter abundances were compared between the three populations.

On the basis of per microgram microsomal proteins, no differences were seen in both CYP3A4 and CYP2C8 enzymes. Thus, significant differences between all three populations for the CYP3A4 enzyme abundance are related to the liver size, as larger liver volumes are seen for virtual subjects with higher BMI values.^18^ Additionally, studies have reported a reduction of 30% and 10% to 40% of CYP3A4 activities in both cancer and obese populations, which further suggests the differences between all three populations.^22,26,27^ There is no specific explanation for the results of no difference between overweight and obese cancer populations for the CYP2C8 enzyme abundance as the lack of information on the CYP2C8 enzyme abundance compared to the CYP3A4 in both cancer and obese populations.^22,26^

For the plasma proteins, studies reported lower human serum albumin (HSA) and higher variability in cancer patients compared to healthy volunteers while unaltered compared to obese subjects.^22,28,29^ Besides, the positive correlation graph between albumin and BMI in cancer patients published by Cheeti et al. (2013)^29^ relates to the notable differences between lean and obese, as well as obese and overweight.

The α1-acid glycoprotein (AGP), another plasma protein component, has been reported to be higher in cancer patients by up to 5-fold compared to the healthy population.^29,30^ Likewise, the AGP level is higher in the obese population than in the healthy population by up to 2.5-fold.^22,31,32^ However, limited information was available for obese cancer patients. Considering AGP is significantly higher in both cancer and obese populations, no difference in the AGP value is anticipated in obese, overweight, and lean cancer patients.

Haematocrit levels in cancer patients are lower than in healthy volunteers but constant throughout the BMI range, clarifying the consistent level between all three populations.^22,29^

# Section 7: Sensitivity analysis for α1-acid glycoprotein (AGP) and CYP3A4 hepatic abundances

In light of the significant influence of CYP3A4 enzyme and AGP level on the metabolism, distribution and elimination of imatinib and the scarcity of information on the CYP3A4 abundance as well as AGP level in obese adult cancer, a sensitivity analysis was performed to explore their impact on the key pharmacokinetic parameters, specifically the C_max_, C_min_ and AUC. Moreover, the results from physiological parameters comparison between the virtual lean, overweight and obese adult cancer populations disclose no difference in both CYP3A4 abundance per microgram microsomal protein and AGP level, reinforcing the necessity for a sensitivity analysis.

The analysis revealed that both the AGP plasma protein binding component and CYP3A4 metabolism enzyme significantly influence all three pharmacokinetic parameters, with the AGP protein exhibiting a more pronounced effect than the CYP3A4 (Figure S6). Notably, the results showed a positive correlation between AGP level and imatinib plasma level concentrations, in contrast to the relationship between CYP3A4 and imatinib concentrations, which aligns with findings in other studies.^33-35^

The result aligns with the fact that imatinib is primarily bound to AGP with high affinity and has been reported to influence imatinib pharmacokinetics substantially.^9,33^ Furthermore, AGP has been linked to imatinib resistance, where studies have shown that AGP plasma concentrations are higher in resistant CML patients and are categorised as one of the resistant mechanisms that are independent of the BCR-ABL gene.^36-38^ Nonetheless, at the same time, the CYP3A4, as the primary isoenzyme involved in imatinib metabolism, contribute considerably to altering the imatinib pharmacokinetics.^2^

A decrease in CYP3A4 activities and an increase in AGP level seen in the obese population is anticipated to increase the imatinib concentrations significantly in the obese cancer population compared to the lean cancer populations.^22^ On the contrary, the imatinib concentration was lower in the obese cancer population.^39^ The opposite pattern may be attributed to the similar changes in CYP3A4 isoenzyme and AGP levels observed in the cancer patient populations, compounded with the high variabilities of both physiological parameters.^26,29^ Thus, the difference in body weight and BSA is potentially the main contributor to the lower imatinib plasma concentration seen in the obese cancer population.^39-42^

# REFERENCES


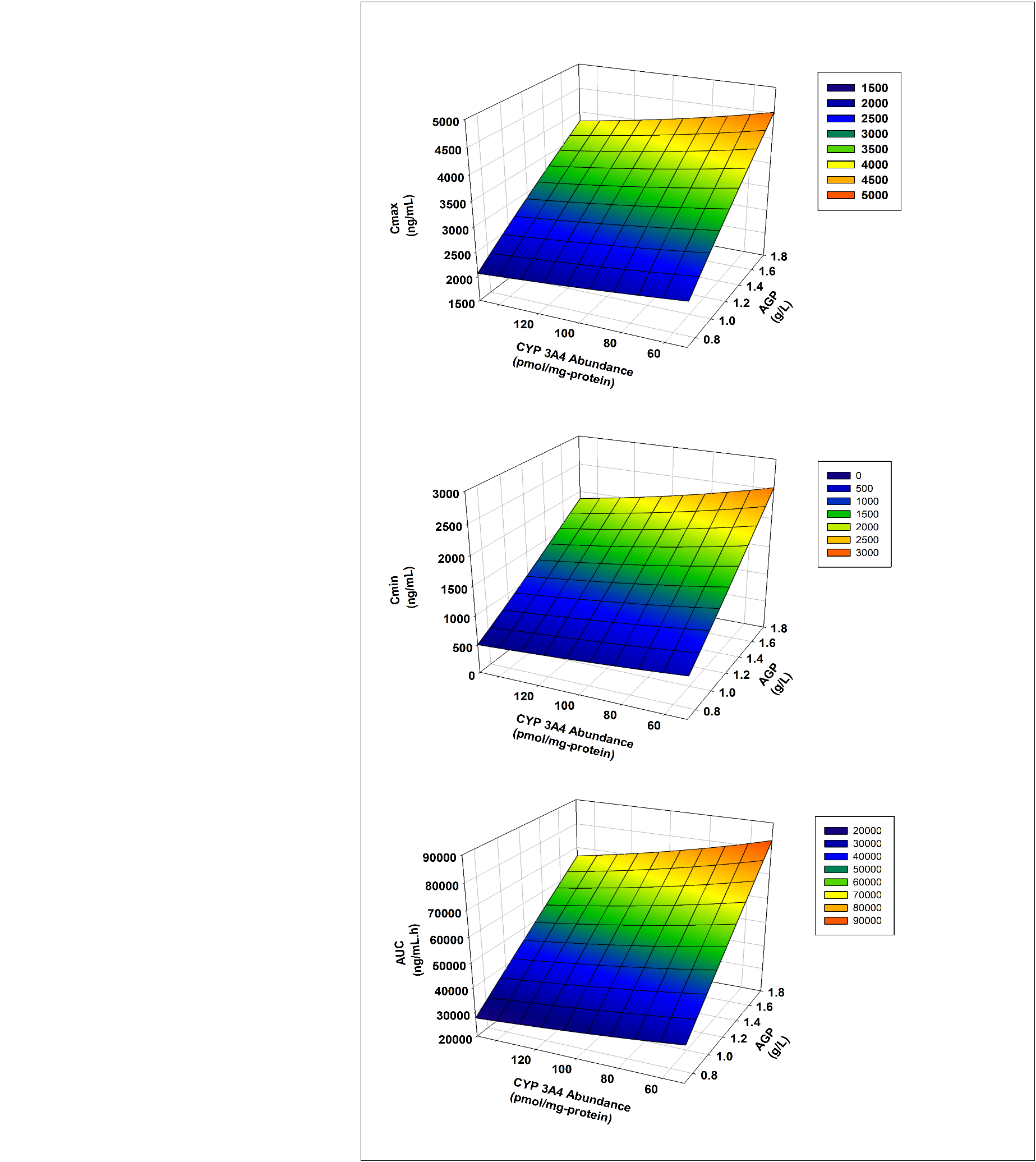


**(A)**

**(B)**

**(C)**

**Figure S6.** Impact of the CYP3A4 hepatic enzyme abundances and AGP changes on the maximum concentration (C_max_) (A), trough concentration (C_min_) (B), and area-under-the-curve (AUC) (C).

1. Peng B, Dutreix C, Mehring G, et al. Absolute bioavailability of imatinib (Glivec) orally versus intravenous infusion. *J Clin Pharmacol*. Feb 2004;44(2):158-62. doi:10.1177/0091270003262101

2. Peng B, Hayes M, Resta D, et al. Pharmacokinetics and pharmacodynamics of imatinib in a phase I trial with chronic myeloid leukemia patients. *J Clin Oncol*. Mar 01 2004;22(5):935-42. doi:10.1200/JCO.2004.03.050

3. Nikolova Z, Peng B, Hubert M, et al. Bioequivalence, safety, and tolerability of imatinib tablets compared with capsules. *Cancer Chemother Pharmacol*. May 2004;53(5):433-8. doi:10.1007/s00280-003-0756-z

4. Pena M, Muriel J, Saiz-Rodríguez M, et al. Effect of Cytochrome P450 and ABCB1 Polymorphisms on Imatinib Pharmacokinetics After Single-Dose Administration to Healthy Subjects. *Clin Drug Investig*. Jul 2020;40(7):617-628. doi:10.1007/s40261-020-00921-7

5. Petain A, Kattygnarath D, Azard J, et al. Population pharmacokinetics and pharmacogenetics of imatinib in children and adults. *Clin Cancer Res*. Nov 01 2008;14(21):7102-9. doi:10.1158/1078-0432.CCR-08-0950

6. Eechoute K, Fransson MN, Reyners AK, et al. A long-term prospective population pharmacokinetic study on imatinib plasma concentrations in GIST patients. *Clin Cancer Res*. Oct 15 2012;18(20):5780-7. doi:10.1158/1078-0432.CCR-12-0490

7. Renard D, Bouillon T, Zhou P, Flesch G, Quinn D. Pharmacokinetic interactions among imatinib, bosentan and sildenafil, and their clinical implications in severe pulmonary arterial hypertension. *Br J Clin Pharmacol*. Jul 2015;80(1):75-85. doi:10.1111/bcp.12584

8. Gotta V, Bouchet S, Widmer N, et al. Large-scale imatinib dose-concentration-effect study in CML patients under routine care conditions. *Leuk Res*. Jul 2014;38(7):764-72. doi:10.1016/j.leukres.2014.03.023

9. Widmer N, Decosterd LA, Csajka C, et al. Population pharmacokinetics of imatinib and the role of alpha-acid glycoprotein. *Br J Clin Pharmacol*. Jul 2006;62(1):97-112. doi:10.1111/j.1365-2125.2006.02719.x

10. Haouala A, Widmer N, Guidi M, et al. Prediction of free imatinib concentrations based on total plasma concentrations in patients with gastrointestinal stromal tumours. *Br J Clin Pharmacol*. Apr 2013;75(4):1007-18. doi:10.1111/j.1365-2125.2012.04422.x

11. Wishart DS, Feunang YD, Guo AC, et al. DrugBank 5.0: a major update to the DrugBank database for 2018. *Nucleic Acids Res*. Jan 4 2018;46(D1):D1074-D1082. doi:10.1093/nar/gkx1037

12. Peng B, Lloyd P, Schran H. Clinical pharmacokinetics of imatinib. *Clin Pharmacokinet*. 2005;44(9):879-94. doi:10.2165/00003088-200544090-00001

13. Kretz O, Weiss HM, Schumacher MM, Gross G. In vitro blood distribution and plasma protein binding of the tyrosine kinase inhibitor imatinib and its active metabolite, CGP74588, in rat, mouse, dog, monkey, healthy humans and patients with acute lymphatic leukaemia. *Br J Clin Pharmacol*. Aug 2004;58(2):212-6. doi:10.1111/j.1365-2125.2004.02117.x

14. Smith P, Bullock JM, Booker BM, Haas CE, Berenson CS, Jusko WJ. The influence of St. John's wort on the pharmacokinetics and protein binding of imatinib mesylate. *Pharmacotherapy*. Nov 2004;24(11):1508-14. doi:10.1592/phco.24.16.1508.50958

15. Rodgers T, Rowland M. Physiologically based pharmacokinetic modelling 2: predicting the tissue distribution of acids, very weak bases, neutrals and zwitterions. *J Pharm Sci*. Jun 2006;95(6):1238-57. doi:10.1002/jps.20502

16. Bornhauser M, Pursche S, Bonin M, et al. Elimination of imatinib mesylate and its metabolite N-desmethyl-imatinib. *J Clin Oncol*. Jun 1 2005;23(16):3855-6; author reply 3857-8. doi:10.1200/JCO.2005.05.246

17. Adiwidjaja J, Boddy AV, McLachlan AJ. Implementation of a Physiologically Based Pharmacokinetic Modeling Approach to Guide Optimal Dosing Regimens for Imatinib and Potential Drug Interactions in Paediatrics. *Front Pharmacol*. 2019;10:1672. doi:10.3389/fphar.2019.01672

18. Johnson TN, Tucker GT, Tanner MS, Rostami-Hodjegan A. Changes in liver volume from birth to adulthood: a meta-analysis. *Liver Transpl*. Dec 2005;11(12):1481-93. doi:10.1002/lt.20519

19. Vasan RS. Cardiac function and obesity. *Heart*. Oct 2003;89(10):1127-9. doi:10.1136/heart.89.10.1127

20. Launay-Vacher V, Oudard S, Janus N, et al. Prevalence of Renal Insufficiency in cancer patients and implications for anticancer drug management: the renal insufficiency and anticancer medications (IRMA) study. *Cancer*. Sep 15 2007;110(6):1376-84. doi:10.1002/cncr.22904

21. Levey AS, Bosch JP, Lewis JB, Greene T, Rogers N, Roth D. A more accurate method to estimate glomerular filtration rate from serum creatinine: a new prediction equation. Modification of Diet in Renal Disease Study Group. *Ann Intern Med*. Mar 16 1999;130(6):461-70. doi:10.7326/0003-4819-130-6-199903160-00002

22. Ghobadi C, Johnson TN, Aarabi M, et al. Application of a systems approach to the bottom-up assessment of pharmacokinetics in obese patients: expected variations in clearance. *Clin Pharmacokinet*. Dec 1 2011;50(12):809-22. doi:10.2165/11594420-000000000-00000

23. Chagnac A, Weinstein T, Korzets A, Ramadan E, Hirsch J, Gafter U. Glomerular hemodynamics in severe obesity. *Am J Physiol Renal Physiol*. May 2000;278(5):F817-22. doi:10.1152/ajprenal.2000.278.5.F817

24. Nebot N, Crettol S, d'Esposito F, Tattam B, Hibbs DE, Murray M. Participation of CYP2C8 and CYP3A4 in the N-demethylation of imatinib in human hepatic microsomes. *Br J Pharmacol*. Nov 2010;161(5):1059-69. doi:10.1111/j.1476-5381.2010.00946.x

25. Oostendorp RL, Buckle T, Beijnen JH, van Tellingen O, Schellens JH. The effect of P-gp (Mdr1a/1b), BCRP (Bcrp1) and P-gp/BCRP inhibitors on the in vivo absorption, distribution, metabolism and excretion of imatinib. *Invest New Drugs*. Feb 2009;27(1):31-40. doi:10.1007/s10637-008-9138-z

26. Schwenger E, Reddy VP, Moorthy G, et al. Harnessing Meta-analysis to Refine an Oncology Patient Population for Physiology-Based Pharmacokinetic Modeling of Drugs. *Clin Pharmacol Ther*. Feb 2018;103(2):271-280. doi:10.1002/cpt.917

27. Rivory LP, Slaviero KA, Clarke SJ. Hepatic cytochrome P450 3A drug metabolism is reduced in cancer patients who have an acute-phase response. *Br J Cancer*. Jul 29 2002;87(3):277-80. doi:10.1038/sj.bjc.6600448

28. Woo J, Chan HS, Or KH, Arumanayagam M. Effect of age and disease on two drug binding proteins: albumin and alpha-1- acid glycoprotein. *Clin Biochem*. Aug 1994;27(4):289-92. doi:10.1016/0009-9120(94)90032-9

29. Cheeti S, Budha NR, Rajan S, Dresser MJ, Jin JY. A physiologically based pharmacokinetic (PBPK) approach to evaluate pharmacokinetics in patients with cancer. *Biopharm Drug Dispos*. Apr 2013;34(3):141-54. doi:10.1002/bdd.1830

30. Paxton JW, Briant RH. Alpha 1-acid glycoprotein concentrations and propranolol binding in elderly patients with acute illness. *Br J Clin Pharmacol*. Nov 1984;18(5):806-10. doi:10.1111/j.1365-2125.1984.tb02548.x

31. Benedek IH, Fiske WD, 3rd, Griffen WO, Bell RM, Blouin RA, McNamara PJ. Serum alpha 1-acid glycoprotein and the binding of drugs in obesity. *Br J Clin Pharmacol*. Dec 1983;16(6):751-4. doi:10.1111/j.1365-2125.1983.tb02258.x

32. Benedek IH, Blouin RA, McNamara PJ. Serum protein binding and the role of increased alpha 1-acid glycoprotein in moderately obese male subjects. *Br J Clin Pharmacol*. Dec 1984;18(6):941-6. doi:10.1111/j.1365-2125.1984.tb02567.x

33. Gambacorti-Passerini C, Zucchetti M, Russo D, et al. Alpha1 acid glycoprotein binds to imatinib (STI571) and substantially alters its pharmacokinetics in chronic myeloid leukemia patients. *Clin Cancer Res*. Feb 2003;9(2):625-32.

34. Dutreix C, Peng B, Mehring G, et al. Pharmacokinetic interaction between ketoconazole and imatinib mesylate (Glivec) in healthy subjects. *Cancer Chemother Pharmacol*. Oct 2004;54(4):290-4. doi:10.1007/s00280-004-0832-z

35. Bolton AE, Peng B, Hubert M, et al. Effect of rifampicin on the pharmacokinetics of imatinib mesylate (Gleevec, STI571) in healthy subjects. *Cancer Chemother Pharmacol*. Feb 2004;53(2):102-6. doi:10.1007/s00280-003-0722-9

36. Larghero J, Leguay T, Mourah S, et al. Relationship between elevated levels of the alpha 1 acid glycoprotein in chronic myelogenous leukemia in blast crisis and pharmacological resistance to imatinib (Gleevec) in vitro and in vivo. *Biochem Pharmacol*. Nov 15 2003;66(10):1907-13. doi:10.1016/s0006-2952(03)00469-6

37. Bhamidipati PK, Kantarjian H, Cortes J, Cornelison AM, Jabbour E. Management of imatinib-resistant patients with chronic myeloid leukemia. *Ther Adv Hematol*. Apr 2013;4(2):103-17. doi:10.1177/2040620712468289

38. Gambacorti-Passerini C, Barni R, le Coutre P, et al. Role of alpha1 acid glycoprotein in the in vivo resistance of human BCR-ABL(+) leukemic cells to the abl inhibitor STI571. *J Natl Cancer Inst*. Oct 18 2000;92(20):1641-50. doi:10.1093/jnci/92.20.1641

39. Lin L, van der Meer EKO, Steeghs N, Beijnen JH, Huitema ADR. Are novel oral oncolytics underdosed in obese patients? *Cancer Chemother Pharmacol*. Oct 31 2023;doi:10.1007/s00280-023-04601-z

40. Horikoshi A, Takei K, Sawada S. Relationship between daily dose of imatinib per square meter and its plasma concentration in patients with chronic-phase chronic myeloid leukemia (CML). *Leuk Res*. Apr 2007;31(4):574-5. doi:10.1016/j.leukres.2006.05.019

41. Park SJ, Choi IK, Seo HY, et al. Reduced dose of imatinib for patients with chronic myeloid leukemia and low body surface area. *Acta Haematol*. 2007;118(4):219-21. doi:10.1159/000111777

42. Breccia M, Molica M, Colafigli G, Alimena G. The Importance of Body Surface Area at Baseline and during Treatment in Chronic Myeloid Leukemia Patients Treated with Imatinib. *Acta Haematologica*. 2015;134(1):57-58. doi:10.1159/000370098
